# Economic evaluation of first-line nivolumab plus cabozantinib for advanced renal cell carcinoma in China

**DOI:** 10.3389/fpubh.2022.954264

**Published:** 2022-09-07

**Authors:** Hao Wang, Ye Wang, Li Li, Han Zhou, Shang Lili, Liao Li, Shen Yike, Ma Aixia

**Affiliations:** ^1^Department of Pharmacoeconomics, School of International Pharmaceutical Business, China Pharmaceutical University, Nanjing, China; ^2^Department of Pharmacy, Nanjing Drum Tower Hospital, Nanjing, China; ^3^Department of Clinical Pharmacy, School of Basic Medical Sciences and Clinical Pharmacy, China Pharmaceutical University, Nanjing, China

**Keywords:** cost-effectiveness, nivolumab, cabozantinib, partitioned survival, renal cell carcinoma

## Abstract

**Background:**

In the Checkmate9ER trial, first-line treatment with nivolumab combined with cabozantinib (NI + CA) has shown efficacy for advanced renal cell carcinoma. This study aims to evaluate the impact of the health and economic outcomes of NI + CA in China.

**Methods:**

Clinical efficacy data were derived from pivotal phase III CheckMate 9ER trial. A three-state partitioned survival model was established based on disease progression. Progression-free survival and overall survival of NI + CA vs. sunitinib were fitted with log-logistic and log-normal distributions, respectively. Mixture cure, non-mixture cure, and Royston/Parmar spline models were used to evaluate model robustness. The results derived the computational cost from the Chinese healthcare system perspective. The primary outcomes were quality-adjusted life-years (QALYs), total cost in US dollars, as well as incremental cost-effectiveness ratios (ICERs) at the willingness-to-pay threshold in China. One-way and probabilistic sensitivity analysis were also used to assess the robustness of the model.

**Results:**

In the base-case analysis result, 0.86 additional QALYs could be obtained in the NI+CA (3.84 QALYs) versus the sunitinib strategy (2.97 QALYs). The ICER of NI+CA compared with the sunitinib strategy was US$292,945 per QALY. The ICER value in the NI+CA strategy was higher than the Chinese willingness-to-pay threshold of US$38,024 per QALY. Although NI+CA can improve long-term patient survival significantly over sunitinib in the treatment of advanced renal cell carcinoma, it is unlikely to be cost-effective due to high cost. The results of the one-way sensitivity analysis showed that drug cost, health utility value at the stage of disease progression, and subsequent treatment proportion had a greater impact on the stability of ICER values.

**Conclusions:**

Nivolumab combined with cabozantinib can prolong the life of patients with advanced renal cell carcinoma and improve their quality of life, but there is a corresponding increase in medical cost. The NI + CA strategy is unlikely to be considered cost-effective in the treatment of advanced RCC from the perspective of Chinese healthcare system.

## Introduction

Renal cell carcinoma (RCC) is a common form of cancer, accounting for 2%−3% of all cancers globally, and has shown an increasing trend over the past decade (1–3). According to global cancer statistics, the annual incidence and mortality rates of kidney cancer during 2020 in China were ~66,800 and 23,400, respectively (4). The number of disability-adjusted life years caused by renal cancer in China is as high as 643,000 years, accounting for 0.17% of the total disability-adjusted life years (5). This disease poses a severe economic burden and public health problem, especially for countries with limited health resources (6).

Anti-angiogenic therapy with sunitinib, a small molecule tyrosine kinase inhibitor, has historically been an effective tool for the first-line treatment of patients with RCC characterized by the inactivation or deletion of the von Hippel-Lindau (VHL) gene (7, 8). Sunitinib has been approved by the Food and Drug Administration as a first-line treatment for advanced and/or metastatic RCC (mRCC). The Guidelines of the Chinese Society of Clinical Oncology (CSCO) for Kidney Cancer includes the first-line sunitinib treatment as a category 1A recommended regimen for patients with mRCC across all risk groups (9). In evaluating the cost-effectiveness of mRCC treatment, sunitinib has always been a strong standard in line with the principles in the China Guidelines for Pharmacoeconomic Evaluations 2020 (10). However, it has now been replaced by treatment with different combinations of immune checkpoint inhibitors (ICIs), kinase inhibitors, and signal transduction blockers based on multiple randomized controlled trials (RCTs) (11–15). Nivolumab is a monoclonal antibody developed against PD-1 that has considerable clinical benefits and an acceptable safety profile for a variety of tumor types (16). Cabozantinib is a tyrosine kinase that has shown efficacy in the CABOSUN RCT and is used as monotherapy for advanced RCC (17). Recently, in a phase-III clinical trial, CheckMate 9ER, nivolumab combined with cabozantinib (NI + CA) showed clear safety and clinical activity in the first-line treatment of advanced RCC with clear histological features. The trial included 651 patients in 18 countries over 20 months. NI+CA significantly improved overall survival (OS), progression-free survival (PFS), and health-related quality of life (QOL) compared with the sunitinib strategy. The PFS was 16.6 months for the NI+CA strategy and 8.3 months for the sunitinib one (median, 16.6 vs. 8.3 months; HR, 0.51; 95% CI, 0.41–0.64). The OS probability at 12 months was 85.7% for the NI+CA strategy and 75.6% for the sunitinib one (HR, 0.60; 98.89% CI, 0.40 to 0.89; *P* = 0.001).

Based on this study, in 2021, the American Society of Clinical Oncology and the Chinese Society of Clinical Oncology recommended NI+CA as a substitute for first-line treatment of advanced RCC. The dual combination of ICIs and kinase inhibitors improves health outcomes in patients with advanced RCC. However, the two-drug combination generates higher medical costs than the ICI regimen, placing a higher economic burden on health insurance finances (18, 19). At present, there is no pharmacoeconomic evaluation of NI + CA strategy in patients with advanced RCC from the perspective of Chinese healthcare system. We thus compare the cost-effectiveness of the NI + CA strategy over sunitinib strategy to treat advanced RCC by using model data from CheckMate9ER. The findings provide evidence for use by patients with advanced first-line RCC and the physicians treating them, as well as health policymakers.

### Model overview

**Figure 1:**
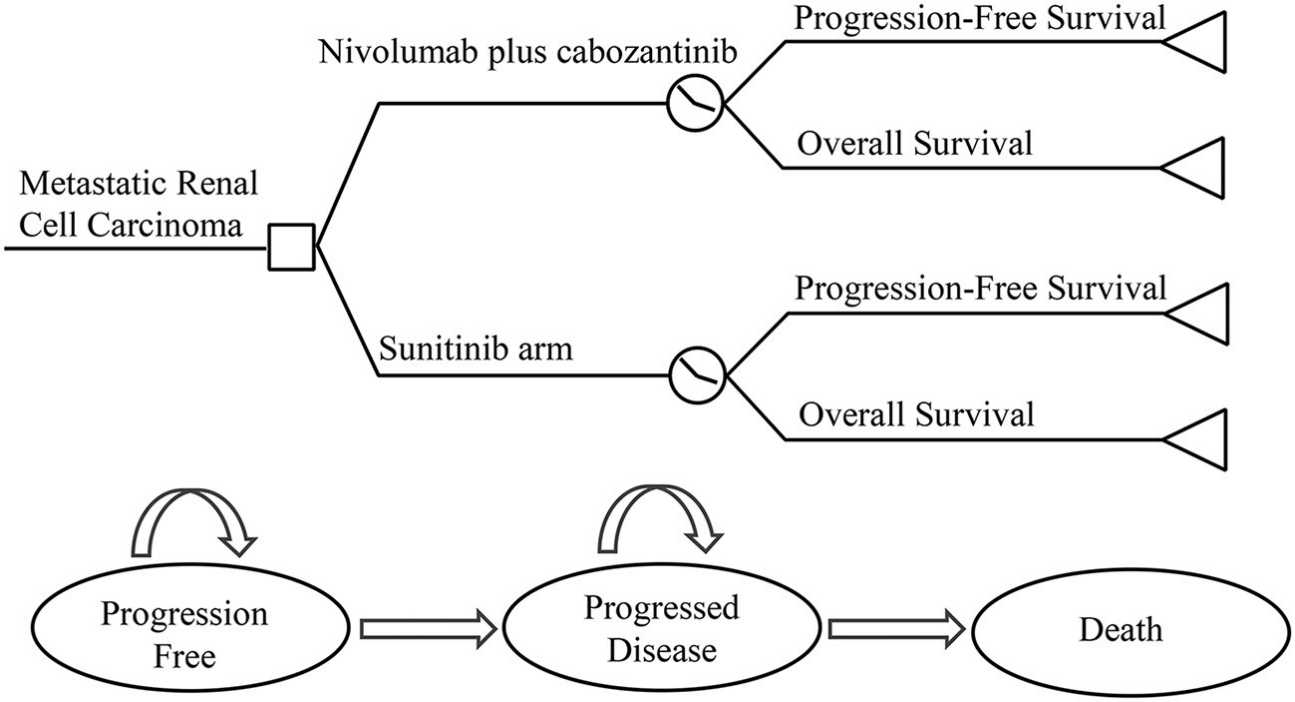
Model structure.

A Treeage ProSuit 2020 was used to construct a three-state partitioned survival (PS) model to assess the economic benefits of NI + CA vs. sunitinib for first-line treatment of RCC from the perspective of the China health system. The model was constructed using a partitioned survival model, an approach widely used in health technology assessment to simulate disease progression and death in advanced RCC and other tumor indications (20, 21). A standard three-state partitioned survival model was employed (see Figure 1), with state membership determined by survival curves. The model cycle was 6 weeks and the study duration 20 years. The model mainly calculates direct medical costs and the adverse event rate was taken from the CheckMate9ER RCT study. Utility values were derived from previous studies (22). According to the China Guidelines for Pharmacoeconomic Evaluation issued by the Chinese Pharmaceutical Association, we discounted the cost and utility values by 5% per year. Three-times national GDP per capita in 2021 was used as the willingness-to-pay threshold (US$38,024 per QALY) (23). The results of the model were expressed as total cost, quality-adjusted life-years (QALYs), and incremental cost-effectiveness ratio (ICER), being calculated using the January 2022 bank foreign exchange rate (US$1 vs. RMB6.3746). Because the economic evaluation was based on a literature review and experimental models, approval from an institutional review board or ethics committee was not required.

### Clinical data

The inclusion criteria and treatment regimen for the study target population were obtained from the CheakMate9ER clinical trial (8). This study included 638 patients with a median follow-up time of 18.1 months. The included patients all had pathologically diagnosed RCC. They received one of the following two treatments at the start of the model: oral cabozantinib 40 mg/day in combination with intravenous nivolumab 240 mg/2 weeks, or oral sunitinib 50 mg/day for 4 weeks, followed by 2 weeks off. Both treatments were administered over a 42-day cycle. According to the CheckMate9ER trial, 86.1% of patients received VEGF receptor inhibitors, including axitinib, sunitinib, and pazopanib, after failure of first-line NI+CA therapy, and 73.6% of the patients in the sunitinib group received PD-L1 inhibitors for subsequent treatment, including nivolumab and pembrolizumab. ICIs were used for a maximum of 2 years during treatment. Patients who had not yet received subsequent treatment received only supportive care for the simplicity of the model.

### Curve fit and progression risk estimates

**Figure 2:**
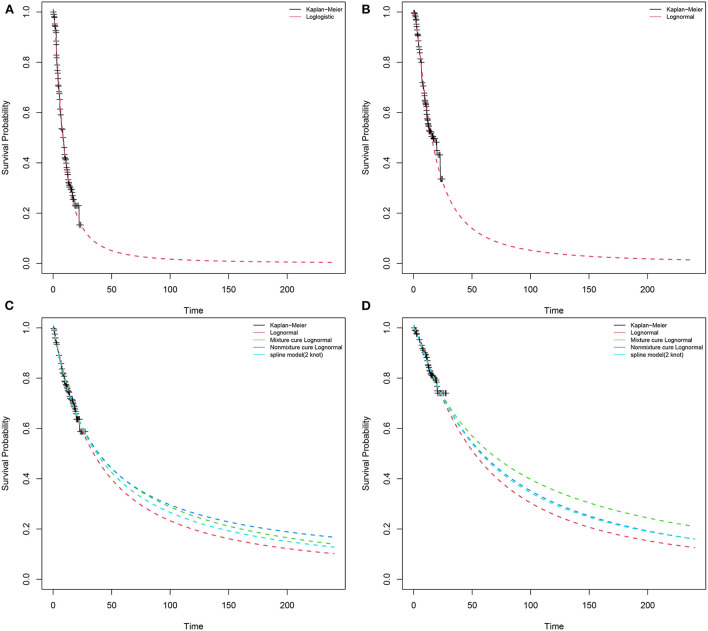
Results of the survival curve fit the NI + CA and sunitinib strategy of the base-case analysis in the partitioned survival model. **(A)** PFS of sunitinib strategy, **(B)** PFS of NI + CA strategy, **(C)** OS of sunitinib strategy, **(D)** OS of NI + CA strategy.

We used Engauge Digitizerversion (https://github.com/markummitchell/engauge-digitizer) and extracted data points from the survival curve in the CheckMate9ER trial. According to Liu et al. (24, 25), individual patients data were reconstructed using the survHE package in R language (v4.1.2) combining KM curve information with the number at risk of events. Exponential, Weibull, log-logistic, log-normal, Gompertz, gen-gamma, Royston/Parmar spline model, and parametric mixture and non-mixture cure models were used to fit distributions to the reconstructed individual patients data (Supplementary Table S1). We compared the reconstructed KM curves with the model extrapolated survival curves (Figure 2). Through visual inspection and comparison with the PFS and OS in the original report, the optimal fitting distribution was judged according to Akaike information criterion (AIC) and Bayesian information criterion (BIC). Finally, log-logistic and log-normal distribution models were chosen to fit the data extracted from the survival curves of PFS for sunitinib and NI + CA, respectively. The log-normal distribution model was selected to fit the OS survival curves of the two groups. There is a plateau at the end of the patient survival curve and there may be an underestimation of survival by traditional parametric models (26). The Royston/Parmar spline, mixture cure, and non-mixture cure models were used to evaluate the robustness of the model.

### Medical costs

In this study, only direct medical costs were considered, including drug treatment, adverse event management, follow-up, and hospital service item costs. The bid prices of drug costs were obtained from the China Pharmaceutical Information Network (www.menet.com). Among the considered drugs, cabozantinib has not been launched in China. The unit prices of cabozantinib were derived from the Centers for Medicare and Medicaid Services (CMS) (27), which belongs to an official government organization in the United States. The follow-up and hospital service item costs mainly included diagnosis, nursing, hospitalization, and intravenous infusion fees, as well as management, electrocardiogram, routine blood, biochemistry, blood coagulation, tumor marker, and enhanced computed tomography costs. Patient follow-up fees and charging standards for hospital service items came from Nanjing Drum Tower Hospital, the Affiliated Hospital of Nanjing University, Jiangsu Province. This model assumes that the average patient weight is 65 kg with a body surface area of 1.72 m^2^. Grade 3–5 adverse events with an incidence above 5% during ICI use should not be ignored. This study derived adverse events (AE) cost partly from the literature (28). We also captured the cost of AE by administering a questionnaire to clinical experts. Table 1 provides detailed information about the costs.

**Table 1:** Summary of main medical costs, utility values, and other parameters.

**Parameter**	**Base case**	**Range**		**Distribution**	**Source**
		**Low**	**High**		
**Clinical inputs**					
Survival model of sunitinib					
Log-logistic model of PFS	Shape, 1.6417 (SE, 0.0976); scale, 8.4452 (SE, 0.5531); AIC, 1,316				
Log-normal model of OS	Meanlog, 3.6682 (SE, 0.1574); SDlog, 1.6679 (SE, 0.1349); AIC, 1,316				
Survival model of NI + CA					
Log-normal model of PFS	Meanlog, 2.1406 (SE, 0.0686); SDlog, 1.0623 (SE, 0.0569); AIC, 709				
Log-normal model of OS	Meanlog, 4.1874 (SE, 0.2046); SDlog, 1.5868 (SE, 0.1603); AIC, 958				
**Drug cost (US$)**					
Nivolumab 100 mg	1,451.07	1,160.85	1,741.28	Gamma	MENET
Cabozantinib 60 mg	491.30	393.04	589.56	Gamma	(23)
Pembrolizumab 100 mg	2,810.84	2,248.67	3,373.01	Gamma	MENET
Sunitinib per table	15.37	12.29	18.44	Gamma	MENET
Axitinib per table	30.85	24.67	37.02	Gamma	MENET
Pazopanib per table	25.10	20.08	30.12	Gamma	MENET
Follow-up cost/cycle (US$)	72.48	57.98	86.97	Gamma	Local market
Management cost/cycle (US$)	46.43	37.15	55.72	Gamma	Local market
Supportive care per cycle (US$)	315.18	282	423	Gamma	(29)
Terminal care (US$)	1,893	946.5	2,839.5	Gamma	(29)
**Cost of managing adverse events (US$)**					
Diarrhea	43.30	34.64	51.96	Gamma	(29)
Hypertension	12.61	10.09	15.14	Gamma	(29)
ALT	25.09	20.07	30.12	Gamma	(29)
Proteinuria	121.65	97.32	145.98	Gamma	(29)
Palmar	102.21	81.77	122.65	Gamma	(29)
**Subsequent treatment proportion**					
Nivolumab + cabozantinib	0.86	0.77	0.94	Beta	(8)
Sunitinib	0.73	0.66	0.80	Beta	(8)
**Risk of adverse events (grade III–IV)**					
Sunitinib	0.75	0.602	0.903	Beta	(8)
Nivolumab + cabozantinib	0.71	0.564	0.847	Beta	(8)
**Health utility**					
Nivolumab + cabozantinib Stable disease	0.82	0.73	0.90	Beta	(20)
Sunitinib stable disease	0.73	0.657	0.803	Beta	(23)
Disease progression	0.66	0.726	0.594	Beta	(23)
Disutility due to AEs (grade ≥3)	0.157	0.127	0.188	Beta	(23)
Discount rate	0.05	0.00	0.08	Fixed in PSA	

### Utility values

Health utility values were obtained from the literature. We assumed a PFS status utility of 0.82 for nivolumab plus cabozantinib, a PFS utility of 0.73 for sunitinib, and a PD status utility of 0.66 (22). Our model included the ≥3-grade treatment related to AE with an incidence above 5%, as reported in the CheckMate9ER trial.

### Sensitivity analysis

To test the robustness of the model, one-way sensitivity analysis and probabilistic sensitivity analysis (PSA) were performed on the parametric model. In the one-way sensitivity analysis, the independent effect of the changes in each parameter on the results was considered. The upper and lower limits of the input were derived from the literature. If the upper and lower 95% CI changes were not available, in which the parameter of cost is in the range of ±20%, the AE incidence and health utility value was designated as ±10%. A reasonable range of discount rate is 0–8%. In the probabilistic sensitivity analyses, a Monte Carlo simulation of 5,000 iterations was generated by simultaneously sampling the key model parameters from the prespecified distributions. A gamma distribution was set for cost parameters and a beta distribution for utility values parameters. The results were shown as a scatter diagram and a cost-effectiveness acceptable curve.

### Scenario analysis

We consider three possible scenario analysis. This study also used scenario analysis to consider the partitioned survival model time extrapolation. The price reduction magnitudes of first-line NI + CA were used to assess their impact on ICER. The approach to the simulated distribution of the Royston/Parmar spline or the non-mixture cure models differ from the standard parametric model. During extrapolation, different distributions of survival models often diverge, often resulting in variations in mean survival and cost-effectiveness estimates. The mixture cure, non-mixture cure, and Royston/Parmar spline models were used to evaluate model robustness.

## Results

### Base-case analysis

The model predicted that the expected result of the NI + CA strategy (3.84 QALYs) was superior to that of the sunitinib strategy (2.97 QALYs) to obtain 0.86 QALYs, but the corresponding cost was US$252,943 greater, resulting in an ICER of US$292,945 per QALY. The results of the base-case analysis are presented in Table 2.

**Table 2:** Results of base-case and scenario analysis.

**Strategy**	**Total cost$**	**Incr cost$**	**LY**	**QALY**	**Incr Eff**	**ICER$/QALYs**
**Base-case analysis**						
Sunitinib	105,820	NA	4.41	2.97	NA	NA
Nivolumab + cabozantinib	358,764	252,943	5.34	3.84	0.86	292,945
**Scenario 1**						
5 years						
Sunitinib	75,520	NA	2.80	1.90	NA	NA
Nivolumab + cabozantinib	316,594	241,073	3.26	2.41	0.51	473,856
10 years						
Sunitinib	92,640	NA	3.71	2.51	NA	NA
Nivolumab + cabozantinib	340,961	248,321	4.45	3.23	0.72	343,900
15 years						
Sunitinib	101,064	NA	4.16	2.81	NA	NA
Nivolumab + cabozantinib	352,484	251,421	5.02	3.62	0.82	307,894
**Scenario 2**						
Adjust nivolumab + cabozantinib 75% of its original price in the first-line setting						
Sunitinib	99,916	NA	4.41	2.97	NA	NA
Nivolumab + cabozantinib	290,413	190,498	5.34	3.84	0.86	220,623
Adjust nivolumab + cabozantinib 50% of its original price in the first-line setting						
Sunitinib	94,011	NA	4.41	2.97	NA	NA
Nivolumab + cabozantinib	222,063	128,052	5.34	3.84	0.86	148,302
Adjust nivolumab + cabozantinib 25% of its original price in the first-line setting.						
Sunitinib	88,106	NA	4.41	2.97	NA	NA
Nivolumab + cabozantinib	153,712	65,606	5.34	3.84	0.86	75,981
**Scenario 3**						
Distribution of OS using parametric survival model						
Sunitinib	105,820	NA	4.41	2.97	NA	NA
Nivolumab + cabozantinib	358,764	252,943	5.34	3.84	0.86	292,945
Distribution of OS using mixture cure model						
Sunitinib	115,260	NA	4.91	3.30	NA	NA
Nivolumab + cabozantinib	375,157	259,897	6.19	4.40	1.10	235,788
Distribution of OS using nonmixture cure model						
Sunitinib	117,410	NA	5.02	3.38	NA	NA
Nivolumab + cabozantinib	366,762	249,352	5.75	4.11	0.74	337,891
Distribution of OS using Royston/Parmar spline model						
Sunitinib	111,675	NA	4.72	3.18	NA	NA
Nivolumab + cabozantinib	365,818	254,143	5.71	4.08	0.90	281,321

LY, life year; QALY, quality-adjusted life-year; ICER, incremental cost-effectiveness ratio.

### Sensitivity analysis

**Figure 3:**
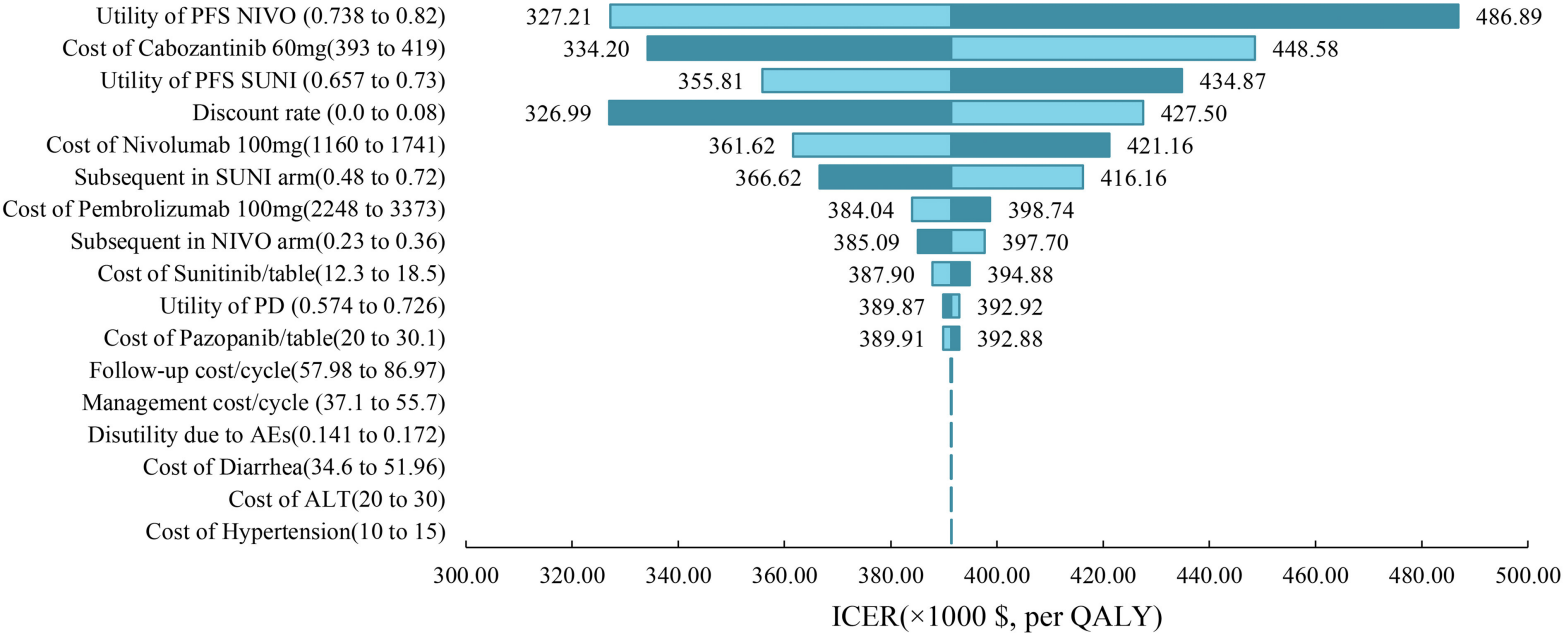
Tornado diagrams showing the effect of lower and upper values of each parameter on the ICERs of the NI + CA vs. sunitinib strategy.

**Figure 4:**
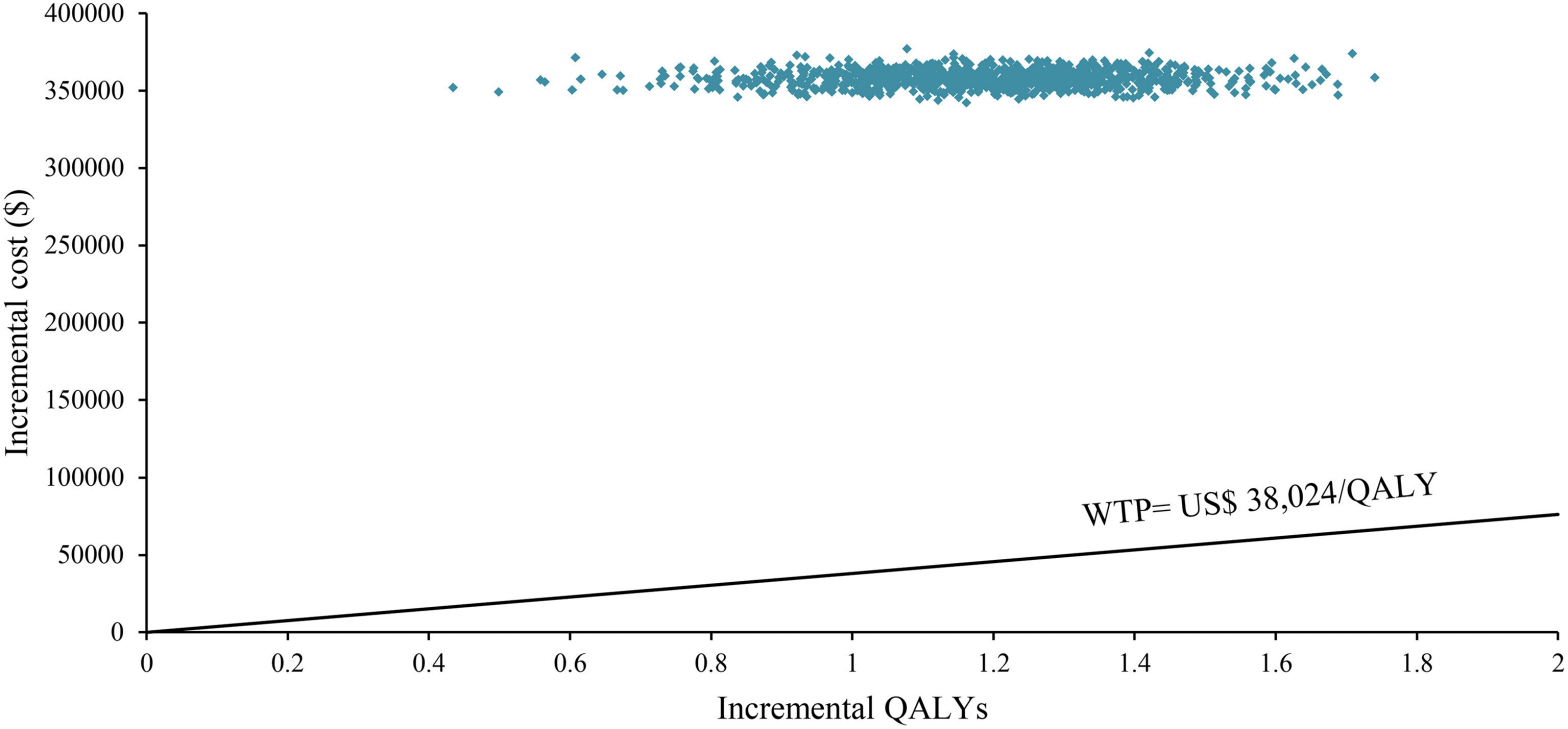
The scatter diagram in the partitioned survival model.

**Figure 5:**
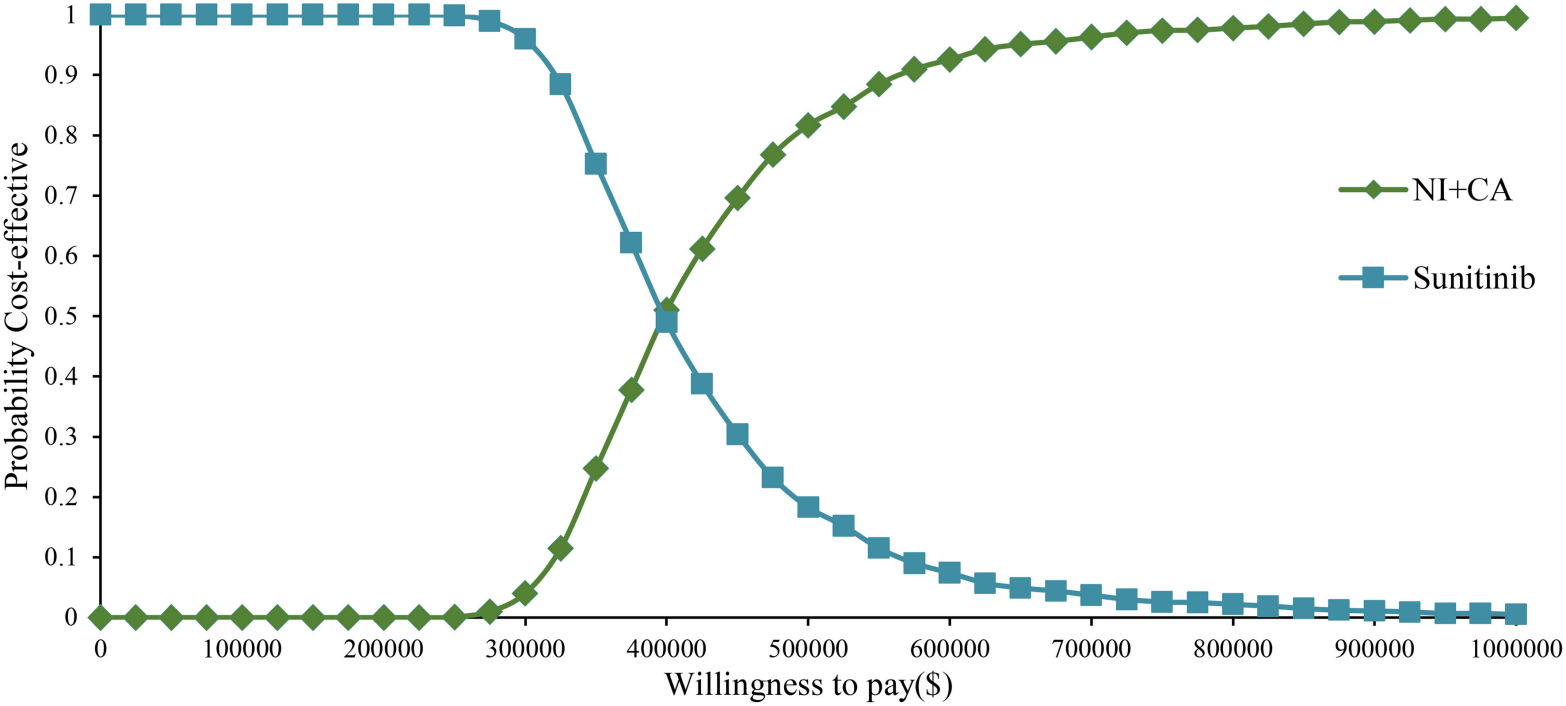
Cost-effectiveness acceptability curve in the partitioned survival model.

One-way sensitivity analysis was represented by a tornado diagram (see Figure 3). The ICER constantly changes when we change the value of each individual by estimating it within a reasonable range. When comparing the NI + CA strategy, the most significant effect on the entire model was the utility value at the PFS stage, followed by the price of cabozantinib. The ICER value changed from US$243,662 to US$367,217 per QALY, being well above US$38,024 per QALY. The other model parameters had a moderate or negligible effect on the expected ICER. When the key model parameters were specifically distributed in the probabilistic sensitivity analysis of NI + CA vs. sunitinib, none of the NI + CA strategies were cost-effective in the Monte Carlo simulations with 5,000 iterations. The scatter diagram revealed the probability of an NI + CA strategy not being a cost-effective option when compared with the sunitinib strategy at a willingness-to-pay threshold of US$38,024/QALY (see Figure 4). The cost-effectiveness acceptability curve reveals the acceptability of NI + CA at different willingness-to-pay threshold (see Figure 5). Compared with sunitinib, NI + CA patients had 0, 60, and 95% probabilities of being cost-effective at patient thresholds above US$100,000, 300,000, and 500,000 per QALY, respectively.

### Scenario analysis

The scenario analysis can be conducted to assess the variability resulting from differences in regions and settings (Table 2). When the model extrapolated with years changing to 5, 10, and 15 years, an interesting phenomenon occurred, with 80% of the medical costs of patients spent in the first 5 years, after which there was still a clinical benefit. In the second scenario analysis, when the purchase price of NI + CA was reduced to 25, 50, and 75%, the ICERs of NI+CA compared with sunitinib were US$75,981, 148,302, and 220,623 per QALY, respectively. The mixture cure model predicted 4.40 and 3.30 QALYs for NI+CA and sunitinib, respectively, with an ICER of US$235,788 per QALY. The non-mixture cure model predicted 4.11 and 3.38 QALYs for NI+CA and sunitinib, respectively, with an ICER of US$337,891 per QALY. The Royston/Parmar spline model predicted 4.08 and 3.18 QALYs for NI + CA and sunitinib, respectively, with an ICER of US$281,321 per QALY.

## Discussion

The high cost of ICIs has always been a hindrance to the use of immunotherapy worldwide, especially where health resources are lacking or are unevenly distributed (29). In the CheckMate9ER study, the combination of immunotherapy and targeted therapy resulted in sustained clinical benefits of improving the QOL of patients with RCC (14). It also places a heavy medical expenditure burden on the financial expenditure of health insurance, especially compared with tyrosine kinase inhibitor monotherapy (18, 22). However, the cost-effectiveness analysis of NI+CA has not been conducted in China. Therefore, we used digital software to reproduce the safety and efficacy data in NI+CA and proposed a model design for the two medication strategies to assess their cost-effectiveness in first-line RCC strategy for long-term extrapolation more than for follow-up cycles. Our results provide important information that can assist in the development of clinical guidelines for the practice of medically treatable treatments based on resource availability.

The willingness-to-pay threshold adopted in this study was three times national GDP per capita (US$38,024 per QALY) in China in 2021, according to the China Guidelines from Pharmacoeconomic Evaluation and World Health Organization standards (23, 30). Based on the results of our model, the ICER of NI + CA with sunitinib was US$391,391.06 per QALY higher than our assumed willingness-to-pay threshold of US$38,024 per QALY. The disadvantage caused by such a huge gap in costs cannot be compensated for by its clinical production. From an economic viewpoint, sunitinib remains the primary option for advanced RCC patients in China with limited health resources. One-way sensitivity analysis indicated that the essential input parameters driving this model were the utility value at the PFS stage and the cost of cabozantinib. Therefore, the most realistic means of proportional cost to clinical value is to reduce the price of cabozantinib and nivolumab, while other nursing treatments can also be adopted in addition to drug therapy to improve the QALYs of patients with increased growth. Contrary to our expectations, cabozantinib had a higher impact on model ICER values than nivolumab. After the NI + CA group's utility value in the PFS stage, the drug-acquisition cost had the second greatest impact in our model. ICIs (pembrolizumab, nivolumab) are the drugs of choice over tyrosine kinase inhibitors in the subsequent treatment with the sunitinib strategy, which leads to a reduced impact of nivolumab on the overall cost of the model.

The cost-effectiveness acceptability curve showed the probability of NI + CA being a cost-effective strategy at different willingness-to-pay threshold per additional QALY gained (Figure 4). The NI + CA strategy is unlikely to be considered cost-effective at a willingness-to-pay threshold of US$38,024 per QALY. However, the combination of the NI + CA strategy is valuable in clinical applications, with significant clinical efficacy and good safety. Healthcare systems can reasonably circumvent the economic burden, as patients with RCC who cannot afford the high price of immune drugs will certainly not suffer the burden. Therefore, if the price of the NI + CA strategy was reduced to 50 and 25% of the original price, the ICER would be reduced to US$148,302 and US$75,981 per QALY, respectively. This would yield an ICER well below the baseline outcome. In the scenario analysis, the model cycle was adjusted to explore the NI +CA strategy in clinical practice for different time horizon. The 5-year survival rate of RCC has been an important assessment reflecting the combined value of immunity. Interestingly, more than 80% of the medical costs of the NI+CA strategy are spent in the first 5 years and patients continue to benefit through subsequent survival. The extrapolation time of the model gradually became longer for 5, 10, and 15 years and the ICER value also decreased.

With the application of ICIs, the survival plot showed a significant plateau at the tail end. Compared with traditional standard parameters, it is necessary to apply the mixture cure, non-mixture cure, and Royston/Parmar spline models to reassess the uncertainty of patients' long-term survival (31). The mixed cure model has large heterogeneity and different distributions produce a large change in outcomes. However, the use of the mixture cure and Royston/Parmar spline models brings more survival benefits to patients than the standard parametric model. In the scenario analysis, patients obtained more QALYs using other extrapolation methods. The ICER varied between models, with the lowest ICER of US$235,788 per QALY in the mixed cure model and the highest ICER of US$337,891 per QALY in the non-mixture cure model. However, this did not change the conclusion that NI + CA was not more cost-effective than sunitinib at a willingness-to-pay threshold of US$38,024 per QALY.

Similar to previous findings for ICIs, Li et al. (32) concluded that an ICER of US$508,987 per QALY for NI + CA vs. sunitinib is not economically feasible from the US health system perspective. From the perspective of the Chinese healthcare system, the ICER for the two groups of medication strategies in this study was US$292,945 per QALY. To further reduce the pharmaceutical burden on patients, the Chinese government has issued a series of policies, including establishing domestic generic drugs and the centralized procurement of drugs with quantity as the core. In the promotion of the procurement of drugs and high-value medical consumables with quantity, the average price reduction for the centralized procurement of the first six batches of drugs under this reform is 53%. Immunotherapy has been found to have a beneficial effect in renal cell cancers, suggesting the advantage of immunomodulating therapies over standard treatment. Nivolumab is an important therapeutic agent for Chinese patients with advanced RCC. As such, if nivolumab can successfully enter the catalog of medicines covered by national medical insurance system, the affordability and accessibility of renal cancer immunotherapy will be greatly improved. And for cabozantinib, although the drug has not yet been marketed in China, from the existing study conclusion, cabozantinib is unlikely to be cost-effective compared with other treatment regimens in China at its current price. We recommend that pharmaceutical companies set appropriate prices or charitable drug donation programs based on China's actual situation to give full play to the advantages of cabozantinib efficacy and safety in clinical treatment.

This study has several methodological strengths. First, the model was constructed using the PS model to perform a 20-year lifecycle analysis for RCC patients. The PS model avoids the calculation of transfer probabilities for cohort members by reconstructing individual patient data. This approach facilitates the validation of the model by other investigators (33, 34). Second, we did not simply specify pembrolizumab as a second-line treatment for all groups, explicitly following modeling based on the information published by the CheckMate9ER trial. This means that our calculated drug cost per subsequent cycle is quite in line with the use of a substantial clinical treatment pathway and significantly reduces the bias of the model in actual extrapolations.

This study has several limitations. First, cabozantinib has not been launched in China. The unit price of cabozantinib in our model was derived from CMS in the United States. Although we performed uncertainty analysis of the price parameters for cabozantinib, this study needs to be further validated after the cabozantinib price is available for a future Chinese launch. In the context of health insurance negotiations, the findings of this study have potential implications for pharmaceutical companies to set prices, while providing a reference basis for health insurance decision-making departments to negotiate prices or decide whether to include them in the health insurance catalog. Second, we used efficacy and safety data from the CheckMate9ER trial for model extrapolation and log-logistic and log-normal parameter distributions to fit the long-term survival of patients. As such, the true efficacy of nivolumab in combination with cabozantinib still needs to be tested in a long-term follow-up study. It is necessary to assess the consistency of these simulation results with real-world efficacy. Third, we assumed that patients could not recover from a progressive disease state to a progression-free disease state, which might have overlooked the health recovery of some patients as well as deaths due to comorbidities. Fourth, we used the QOL scores of mRCC for the European population in the literature, which do not truly reflect the data for Chinese patients, among other population groups. This study showed no significant difference in the QOL between Asian and European populations. The robustness of the model would be significantly improved if future health utility analysis of RCC patients with relevant first-line NI + CA could be performed for the Chinese population. Finally, owing to a lack of some head-to-head trials for renal cell cancer, no strategy of the mutual combination of other PD-L1 drugs with tyrosine kinase inhibitors was included in this study.

## Conclusions

According to the base-case and sensitivity analysis results, the NI+CA strategy is unlikely to be considered cost-effective over sunitinib in the treatment of advanced RCC from the perspective of Chinese healthcare system. ICIs and tyrosine kinase inhibitors benefit patients with advanced renal cancer but incur additional costs. Our findings support the efforts to reduce drug prices and enable this treatment to reduce the economic burden on the Chinese healthcare system.

## Data availability statement

The original contributions presented in the study are included in the article/Supplementary material, further inquiries can be directed to the corresponding author.

## Author contributions

Conception and design: HW and YW. Administrative support: MA. Provision of study materials or patients: YW and ZH. Collection and assembly of data: HW, SL, and LL. (V) Data analysis and interpretation: HW, YW, and SY. Manuscript writing and final approval of the manuscript. All authors contributed to the article and approved the submitted version.

## Conflict of interest

The authors declare that the research was conducted in the absence of any commercial or financial relationships that could be construed as a potential conflict of interest.

## Publisher's note

All claims expressed in this article are solely those of the authors and do not necessarily represent those of their affiliated organizations, or those of the publisher, the editors and the reviewers. Any product that may be evaluated in this article, or claim that may be made by its manufacturer, is not guaranteed or endorsed by the publisher.

## References

[1] Buti S Puligandla M Bersanelli M DiPaola R Manola J Taguchi S. Validation of a new prognostic model to easily predict outcome in renal cell carcinoma: the GRANT score applied to the ASSURE trial population. Ann Oncol. (2017) 28:2747–53. 10.1093/annonc/mdx492 28945839 PMC5815563

[2] Gross-Goupil M Kwon T Eto M Ye D Miyake H Seo S. Axitinib versus placebo as an adjuvant treatment of renal cell carcinoma: results from the phase III, randomized ATLAS trial. Ann Oncol. (2018) 29:2371–8. 10.1093/annonc/mdy454 30346481 PMC6311952

[3] Wang Y Zhang Y Wang P Fu X Lin WJ. Circular RNAs in renal cell carcinoma: implications for tumorigenesis, diagnosis, and therapy. Mol Cancer. (2020) 19:1–10. 10.1186/s12943-020-01266-7 33054773 PMC7559063

[4] Chen W Zheng R Baade PD Zhang S Zeng H Bray F. Cancer statistics in China, 2015. CA Cancer J Clin. (2016) 66:115–32. 10.3322/caac.21338 26808342

[5] Murray CJ Aravkin AY Zheng P Abbafati C Abbas KM Abbasi-Kangevari M. Global burden of 87 risk factors in 204 countries and territories, 1990–2019: a systematic analysis for the Global Burden of Disease Study 2019. Lancet. (2020) 396:1223–49. 33069327 10.1016/S0140-6736(20)30752-2 PMC7566194

[6] Wang H Wang Y Gong R Geng Y. Li LJ. Cost-effectiveness of pertuzumab and trastuzumab as a first-line treatment of HER2-positive metastatic breast cancer in China. Ann Palliat Med. (2021) 10:11382–93. 10.21037/apm-21-2412 34872264

[7] Qin S-K Jin J Guo J Wang J-W Zhou F-J Huang Y-R. Efficacy and safety of first-line sunitinib in Chinese patients with metastatic renal cell carcinoma. Future Oncol. (2018) 14:1835–45. 10.2217/fon-2017-0733 29717651

[8] Rossi E Bersanelli M Gelibter AJ Borsellino N Caserta C Doni L. Combination therapy in renal cell carcinoma: the best choice for every patient? Curr Oncol Rep. (2021) 23:1–12. 10.1007/s11912-021-01140-9 34748099 PMC8575734

[9] Motzer RJ Jonasch E Agarwal N Alva A Baine M Beckermann K. Kidney cancer, version 3.2022, NCCN clinical practice guidelines in oncology. J Natl Compr Canc Netw. (2022) 20:71–90. 10.6004/jnccn.2022.0001 34991070 PMC10191161

[10] Liu G HS Wu J Wu J Yang L Li H. China Guidelines for Pharmacoeconomic Evaluations 2020 (Chinese-English Version). Beijing: China market press (2020).

[11] Cella D Grünwald V Escudier B Hammers HJ George S Nathan P. Patient-reported outcomes of patients with advanced renal cell carcinoma treated with nivolumab plus ipilimumab versus sunitinib (CheckMate 214): a randomised, phase 3 trial. Lancet Oncol. (2019) 20:297–310. 10.1016/S1470-2045(18)30778-2 30658932 PMC6701190

[12] Motzer RJ Penkov K Haanen J Rini B Albiges L Campbell MT. Avelumab plus axitinib versus sunitinib for advanced renal-cell carcinoma. N Engl J Med. (2019) 380:1103–15. 10.1056/NEJMoa1816047 30779531 PMC6716603

[13] Bensimon AG Zhong Y Swami U Briggs A Young J Feng Y. Cost-effectiveness of pembrolizumab with axitinib as first-line treatment for advanced renal cell carcinoma. Curr Med Res Opin. (2020) 36:1507–17. 10.1080/03007995.2020.1799771 32697113

[14] Choueiri TK Powles T Burotto M Escudier B Bourlon MT Zurawski B. Nivolumab plus cabozantinib versus sunitinib for advanced renal-cell carcinoma. N Engl J Med. (2021) 384:829–41. 10.1056/NEJMoa2026982 33657295 PMC8436591

[15] Motzer R Alekseev B Rha S-Y Porta C Eto M Powles T. Lenvatinib plus pembrolizumab or everolimus for advanced renal cell carcinoma. N Engl J Med. (2021) 384:1289–300. 10.1056/NEJMoa2035716 33616314

[16] Esfahani K Roudaia L Buhlaiga Na Del Rincon S Papneja N Miller WJ. A review of cancer immunotherapy: from the past, to the present, to the future. Curr Oncol. (2020) 27:87–97. 10.3747/co.27.5223 32368178 PMC7194005

[17] Choueiri TK Hessel C Halabi S Sanford B Michaelson MD Hahn O. Cabozantinib versus sunitinib as initial therapy for metastatic renal cell carcinoma of intermediate or poor risk (Alliance A031203 CABOSUN randomised trial): progression-free survival by independent review and overall survival update. Eur J Cancer. (2018) 94:115–25. 10.1016/j.ejca.2018.02.012 29550566 PMC6057479

[18] Su Y Fu J Du J Wu BJ. First-line treatments for advanced renal-cell carcinoma with immune checkpoint inhibitors: systematic review, network meta-analysis and cost-effectiveness analysis. Ther Adv Med Oncol. (2020) 12:1758835920950199. 10.1177/1758835920950199 32874210 PMC7436799

[19] Watson TR Gao X Reynolds KL Kong CYJ. Cost-effectiveness of pembrolizumab plus axitinib vs nivolumab plus ipilimumab as first-line treatment of advanced renal cell carcinoma in the US. JAMA Netw Open. (2020) 3:e2016144. 10.1001/jamanetworkopen.2020.16144 33052401 PMC7557509

[20] Woods B Sideris E Palmer S Latimer N Soares Oliveira MF. NICE DSU technical support document 19. Partitioned survival analysis for decision modelling in health care: a critical review. Technical Support Document, NICE Decision Support Unit. (2017). Available online at: http://www.nicedsu.org.uk (accessed July 21, 2022).

[21] Woods BS Sideris E Palmer S Latimer N Soares MJ. Partitioned survival and state transition models for healthcare decision making in oncology: where are we now? Value Health. (2020) 23:1613–21. 10.1016/j.jval.2020.08.2094 33248517

[22] Li S Li J Peng L Li Y Wan XJ. Cost-effectiveness of frontline treatment for advanced renal cell carcinoma in the era of immunotherapies. Front Pharmacol. (2021) 12:718014. 10.3389/fphar.2021.718014 34566643 PMC8458866

[23] Yue X Li Y Wu J Guo JJ. Current development and practice of pharmacoeconomic evaluation guidelines for universal health coverage in China. Value Health Reg Issues. (2021) 24:1–5. 10.1016/j.vhri.2020.07.580 33349598

[24] Guyot P Ades A Ouwens MJ. Welton NJJ. Enhanced secondary analysis of survival data: reconstructing the data from published Kaplan-Meier survival curves. BMC Med Res Methodol. (2012) 12:1–13. 10.1186/1471-2288-12-9 22297116 PMC3313891

[25] Liu N Zhou Y Lee JJJ. IPDfromKM: reconstruct individual patient data from published Kaplan-Meier survival curves. BMC Med Res Methodol. (2021) 21:1–22. 10.1186/s12874-021-01308-8 34074267 PMC8168323

[26] Rutherford M Lambert PC Sweeting MJ Pennington R Crowther MJ Abrams KR. Technical support document 21. Flexible Methods for Survival Analysis. Sheffield (2020).

[27] ASP Drug Pricing Files. (2021). Available online at: https://www.cms.gov/Medicare/Medicare-Fee-for-Service-Part-B-Drugs/McrPartBDrugAvgSalesPrice/2021ASPFiles.html (accessed July 25, 2021).

[28] Chen J Hu G Chen Z Wan X Tan C Zeng X. Cost-effectiveness analysis of pembrolizumab plus axitinib versus sunitinib in first-line advanced renal cell carcinoma in China. Clin Drug Investig. (2019) 39:931–8. 10.1007/s40261-019-00820-6 31250401

[29] Ocran Mattila P Ahmad R Hasan SS Babar Z-U-DJ. Availability, affordability, access, and pricing of anti-cancer medicines in low-and middle-income countries: a systematic review of literature. Front Public Health. (2021) 9:462. 10.3389/fpubh.2021.628744 33996712 PMC8120029

[30] Bertram MY Edejer TTT. Introduction to the special issue on “the world health organization choosing interventions that are cost-effective (WHO-CHOICE) update”. Int J Health Policy Manag. (2021) 10:670–2. 10.34172/ijhpm.2021.105 34634892 PMC9278374

[31] Filleron T Bachelier M Mazieres J Pérol M Meyer N Martin E. Assessment of treatment effects and long-term benefits in immune checkpoint inhibitor trials using the flexible parametric cure model: a systematic review. JAMA Netw Open. (2021) 4:e2139573-e. 10.1001/jamanetworkopen.2021.39573 34932105 PMC8693223

[32] Li S Li J Peng L Li Y. Wan XJ. Cost-effectiveness of nivolumab plus cabozantinib versus sunitinib as a first-line treatment for advanced renal cell carcinoma in the United States. Front Pharmacol. (2021) 12:736860. 10.3389/fphar.2021.736860 34966275 PMC8711761

[33] Latimer NRJ. Survival analysis for economic evaluations alongside clinical trials—extrapolation with patient-level data: inconsistencies, limitations, and a practical guide. Med Decis Making. (2013) 33:743–54. 10.1177/0272989X12472398 23341049

[34] Baio GJ. survHE: survival analysis for health economic evaluation and cost-effectiveness modeling. J Stat Softw. (2020) 95:1–47. 10.18637/jss.v095.i14

