# Supplementary material for: Economic evaluation of first-line nivolumab plus cabozantinib for advanced renal cell carcinoma in China
Source: Front Public Health. 2022 Sep 7;10:954264. doi: 10.3389/fpubh.2022.954264 (PMC9490003; doi:10.3389/fpubh.2022.954264)
Supplement: Supplementary file 1 [file Table_1.DOCX]

Supplementary Material

Economic Evaluation of First-Line Nivolumab plus Cabozantinib for Advanced Renal Cell Carcinoma in China

.

**TableS1.** Fit parameters, AIC and BIC values for each model

PFS, progression-free survival; OS, overall survival; AIC, Akaike information criterion; BIC, Bayesian information criterion.

|  |  |  | **NI+CA** | | | | **Sunitinib** | | | |
| --- | --- | --- | --- | --- | --- | --- | --- | --- | --- | --- |
| **Index** | **Distribution** | **Parameter** | **Est** | **Se** | **AIC** | **BIC** | **Est** | **Se** | **AIC** | **BIC** |
| **PFS** | Log-logistic | shape | 1.6417 | 0.0976 | 1316.20 | 1323.79 | 1.6417 | 0.0976 | 1316.20 | 1323.79 |
|  |  | scale | 8.4452 | 0.5531 | 1316.20 | 1323.79 | 8.4452 | 0.5531 | 1316.20 | 1323.79 |
|  | Weibull | shape | 1.2500 | 0.0742 | 1329.56 | 1337.14 | 1.2500 | 0.0742 | 1329.56 | 1337.14 |
|  |  | scale | 12.3277 | 0.7273 | 1329.56 | 1337.14 | 12.3277 | 0.7273 | 1329.56 | 1337.14 |
|  | Log-normal | meanlog | 2.1406 | 0.0686 | 1316.20 | 1323.79 | 2.1406 | 0.0686 | 1316.20 | 1323.79 |
|  |  | sdlog | 1.0623 | 0.0569 | 1316.20 | 1323.79 | 1.0623 | 0.0569 | 1316.20 | 1323.79 |
|  | Gamma | shape | 1.4619 | 0.1286 | 1325.24 | 1332.83 | 1.4619 | 0.1286 | 1325.24 | 1332.83 |
|  |  | rate | 0.1264 | 0.0157 | 1325.24 | 1332.83 | 0.1264 | 0.0157 | 1325.24 | 1332.83 |
|  | Exponential | rate | 0.0773 | 0.0056 | 1340.38 | 1344.17 | 0.0773 | 0.0056 | 1340.38 | 1344.17 |
|  | Gen-gamma | mu | 2.1957 | 0.1099 | 1317.81 | 1329.18 | 2.1957 | 0.1099 | 1317.81 | 1329.18 |
|  |  | sigma | 1.0301 | 0.0767 | 1317.81 | 1329.18 | 1.0301 | 0.0767 | 1317.81 | 1329.18 |
|  |  | Q | 0.1377 | 0.2200 | 1317.81 | 1329.18 | 0.1377 | 0.2200 | 1317.81 | 1329.18 |
|  | Mixture cure model (log-logistic) | theta | 0.0996 | NA | 1316.54 | 1327.92 | 0.0996 | NA | 1316.54 | 1327.92 |
|  |  | shape | 1.7952 | 0.1572 | 1316.54 | 1327.92 | 1.7952 | 0.1572 | 1316.54 | 1327.92 |
|  |  | scale | 7.2677 | 0.8433 | 1316.54 | 1327.92 | 7.2677 | 0.8433 | 1316.54 | 1327.92 |
|  | Mixture cure model (Weibull) | theta | 0.2076 | NA | 1321.98 | 1333.36 | 0.2076 | NA | 1321.98 | 1333.36 |
|  |  | shape | 1.4778 | 0.1024 | 1321.98 | 1333.36 | 1.4778 | 0.1024 | 1321.98 | 1333.36 |
|  |  | scale | 8.5468 | 0.7186 | 1321.98 | 1333.36 | 8.5468 | 0.7186 | 1321.98 | 1333.36 |
|  | Mixture cure model (log-normal) | theta | 0.0076 | NA | 1318.20 | 1329.58 | 0.0076 | NA | 1318.20 | 1329.58 |
|  |  | meanlog | 2.1290 | 0.1536 | 1318.20 | 1329.58 | 2.1290 | 0.1536 | 1318.20 | 1329.58 |
|  |  | sdlog | 1.0565 | 0.0899 | 1318.20 | 1329.58 | 1.0565 | 0.0899 | 1318.20 | 1329.58 |
|  | Mixture cure model (gamma) | theta | 0.1920 | NA | 1318.55 | 1329.93 | 0.1920 | NA | 1318.55 | 1329.93 |
|  |  | shape | 1.8455 | 0.2046 | 1318.55 | 1329.93 | 1.8455 | 0.2046 | 1318.55 | 1329.93 |
|  |  | rate | 0.2289 | 0.0419 | 1318.55 | 1329.93 | 0.2289 | 0.0419 | 1318.55 | 1329.93 |
|  | Nonmixture cure model (log-logistic)) | theta | 0.1223 | NA | 1317.23 | 1328.61 | 0.1223 | NA | 1317.23 | 1328.61 |
|  |  | shape | 1.7412 | 0.1564 | 1317.23 | 1328.61 | 1.7412 | 0.1564 | 1317.23 | 1328.61 |
|  |  | scale | 12.3620 | 2.7319 | 1317.23 | 1328.61 | 12.3620 | 2.7319 | 1317.23 | 1328.61 |
|  | Nonmixture cure model (Weibull) | theta | 0.1877 | NA | 1319.19 | 1330.57 | 0.1877 | NA | 1319.19 | 1330.57 |
|  |  | shape | 1.5993 | 0.1169 | 1319.19 | 1330.57 | 1.5993 | 0.1169 | 1319.19 | 1330.57 |
|  |  | scale | 12.3779 | 2.0410 | 1319.19 | 1330.57 | 12.3779 | 2.0410 | 1319.19 | 1330.57 |
|  | Nonmixture cure model (log-normal) | theta | 0.0178 | NA | 1316.94 | 1328.31 | 0.0178 | NA | 1316.94 | 1328.31 |
|  |  | meanlog | 3.3959 | 0.6816 | 1316.94 | 1328.31 | 3.3959 | 0.6816 | 1316.94 | 1328.31 |
|  |  | sdlog | 1.3393 | 0.2099 | 1316.94 | 1328.31 | 1.3393 | 0.2099 | 1316.94 | 1328.31 |
|  | Nonmixture cure model (gamma) | theta | 0.1637 | NA | 1317.20 | 1328.57 | 0.1637 | NA | 1317.20 | 1328.57 |
|  |  | shape | 1.8747 | 0.2103 | 1317.20 | 1328.57 | 1.8747 | 0.2103 | 1317.20 | 1328.57 |
|  |  | rate | 0.1470 | 0.0429 | 1317.20 | 1328.57 | 0.1470 | 0.0429 | 1317.20 | 1328.57 |
|  | Nonmixture cure model (exp) | theta | 0.0000 | NA | 1342.83 | 1350.42 | 0.0000 | NA | 1342.83 | 1350.42 |
|  |  | rate | 0.0024 | 0.0023 | 1342.83 | 1350.42 | 0.0024 | 0.0023 | 1342.83 | 1350.42 |
|  | Nonmixture cure model (gengamma) | theta | 0.0964 | NA | 1318.34 | 1333.51 | 0.0964 | NA | 1318.34 | 1333.51 |
|  |  | mu | 2.7780 | 0.5333 | 1318.34 | 1333.51 | 2.7780 | 0.5333 | 1318.34 | 1333.51 |
|  |  | sigma | 0.9402 | 0.3230 | 1318.34 | 1333.51 | 0.9402 | 0.3230 | 1318.34 | 1333.51 |
|  |  | Q | 0.3970 | 0.4036 | 1318.34 | 1333.51 | 0.3970 | 0.4036 | 1318.34 | 1333.51 |
|  | Royston/Parmar spline model(0 kont) | gamma0 | -3.5019 | 0.2158 | 1316.20 | 1323.79 | -3.5019 | 0.2158 | 1316.20 | 1323.79 |
|  |  | gamma1 | 1.6414 | 0.0976 | 1316.20 | 1323.79 | 1.6414 | 0.0976 | 1316.20 | 1323.79 |
|  | Royston/Parmar spline model(1 kont) | gamma0 | -3.7807 | 0.2896 | 1315.51 | 1326.89 | -3.7807 | 0.2896 | 1315.51 | 1326.89 |
|  |  | gamma1 | 2.1380 | 0.3329 | 1315.51 | 1326.89 | 2.1380 | 0.3329 | 1315.51 | 1326.89 |
|  |  | gamma2 | 0.0595 | 0.0370 | 1315.51 | 1326.89 | 0.0595 | 0.0370 | 1315.51 | 1326.89 |
|  | Royston/Parmar spline model(2 kont) | gamma0 | -3.7855 | 0.2947 | 1317.52 | 1332.70 | -3.7855 | 0.2947 | 1317.52 | 1332.70 |
|  |  | gamma1 | 2.2028 | 0.5210 | 1317.52 | 1332.70 | 2.2028 | 0.5210 | 1317.52 | 1332.70 |
|  |  | gamma2 | 0.0547 | 0.2222 | 1317.52 | 1332.70 | 0.0547 | 0.2222 | 1317.52 | 1332.70 |
|  |  | gamma3 | 0.0016 | 0.2622 | 1317.52 | 1332.70 | 0.0016 | 0.2622 | 1317.52 | 1332.70 |
|  | Royston/Parmar spline model(3 kont) | gamma0 | -3.8248 | 0.2937 | 1318.29 | 1337.25 | -3.8248 | 0.2937 | 1318.29 | 1337.25 |
|  |  | gamma1 | 1.7845 | 0.5734 | 1318.29 | 1337.25 | 1.7845 | 0.5734 | 1318.29 | 1337.25 |
|  |  | gamma2 | -0.3631 | 0.3468 | 1318.29 | 1337.25 | -0.3631 | 0.3468 | 1318.29 | 1337.25 |
|  |  | gamma3 | 0.9899 | 0.8374 | 1318.29 | 1337.25 | 0.9899 | 0.8374 | 1318.29 | 1337.25 |
|  |  | gamma4 | -0.7250 | 0.6904 | 1318.29 | 1337.25 | -0.7250 | 0.6904 | 1318.29 | 1337.25 |
| **OS** | Log-logistic | shape | 1.2659 | 0.1432 | 709.22 | 716.78 | 1.1238 | 0.1014 | 960.08 | 967.66 |
|  |  | scale | 51.9182 | 8.8082 | 709.22 | 716.78 | 34.6194 | 4.6515 | 960.08 | 967.66 |
|  | Weibull | shape | 1.1839 | 0.1362 | 709.82 | 717.37 | 1.0192 | 0.0946 | 961.53 | 969.11 |
|  |  | scale | 62.2215 | 11.0411 | 709.82 | 717.37 | 45.4307 | 6.2668 | 961.53 | 969.11 |
|  | Log-normal | meanlog | 4.1874 | 0.2046 | 709.10 | 716.65 | 3.6682 | 0.1574 | 958.53 | 966.11 |
|  |  | sdlog | 1.5868 | 0.1603 | 709.10 | 716.65 | 1.6679 | 0.1349 | 958.53 | 966.11 |
|  | Gamma | shape | 1.2329 | 0.1695 | 709.61 | 717.17 | 1.0399 | 0.1175 | 961.45 | 969.04 |
|  |  | rate | 0.0198 | 0.0058 | 709.61 | 717.17 | 0.0232 | 0.0054 | 961.45 | 969.04 |
|  | Exponential | rate | 0.0127 | 0.0016 | 709.84 | 713.61 | 0.0216 | 0.0022 | 959.57 | 963.36 |
|  | Gen-gamma | mu | 4.1933 | 0.1984 | 710.84 | 722.17 | 3.7000 | 0.1923 | 960.46 | 971.84 |
|  |  | sigma | 1.3359 | 0.5021 | 710.84 | 722.17 | 1.5866 | 0.3415 | 960.46 | 971.84 |
|  |  | Q | 0.3222 | 0.6124 | 710.84 | 722.17 | 0.1218 | 0.4652 | 960.46 | 971.84 |
|  | Mixture cure model (log-logistic) | theta | 0.4396 | NA | 710.71 | 722.04 | 0.3300 | NA | 961.33 | 972.71 |
|  |  | shape | 1.4280 | 0.2708 | 710.71 | 722.04 | 1.2633 | 0.1922 | 961.33 | 972.71 |
|  |  | scale | 25.9601 | 17.3815 | 710.71 | 722.04 | 19.0926 | 9.8364 | 961.33 | 972.71 |
|  | Mixture cure model (Weibull) | theta | 0.6453 | NA | 710.50 | 721.83 | 0.4971 | NA | 961.90 | 973.28 |
|  |  | shape | 1.4165 | 0.2364 | 710.50 | 721.83 | 1.1773 | 0.1512 | 961.90 | 973.28 |
|  |  | scale | 19.4620 | 8.4547 | 710.50 | 721.83 | 17.5743 | 6.9424 | 961.90 | 973.28 |
|  | Mixture cure model (log-normal) | theta | 0.0043 | NA | 711.10 | 722.44 | 0.0024 | NA | 960.53 | 971.91 |
|  |  | meanlog | 4.1828 | 0.2462 | 711.10 | 722.44 | 3.6641 | 0.2036 | 960.53 | 971.91 |
|  |  | sdlog | 1.5856 | 0.1650 | 711.10 | 722.44 | 1.6666 | 0.1412 | 960.53 | 971.91 |
|  | Mixture cure model (gamma) | theta | 0.5792 | NA | 710.64 | 721.97 | 0.4870 | NA | 961.59 | 972.97 |
|  |  | shape | 1.4884 | 0.3345 | 710.64 | 721.97 | 1.2545 | 0.2134 | 961.59 | 972.97 |
|  |  | rate | 0.0645 | 0.0478 | 710.64 | 721.97 | 0.0716 | 0.0376 | 961.59 | 972.97 |
|  | Nonmixture cure model (log-logistic)) | theta | 0.4475 | NA | 710.70 | 722.03 | 0.3445 | NA | 961.38 | 972.76 |
|  |  | shape | 1.4249 | 0.2709 | 710.70 | 722.03 | 1.2579 | 0.1923 | 961.38 | 972.76 |
|  |  | scale | 33.6299 | 29.6684 | 710.70 | 722.03 | 27.9198 | 20.3908 | 961.38 | 972.76 |
|  | Nonmixture cure model (Weibull) | theta | 0.6238 | NA | 710.57 | 721.90 | 0.4711 | NA | 961.67 | 973.05 |
|  |  | shape | 1.4260 | 0.2461 | 710.57 | 721.90 | 1.2054 | 0.1594 | 961.67 | 973.05 |
|  |  | scale | 23.3798 | 14.2654 | 710.57 | 721.90 | 23.3591 | 13.9729 | 961.67 | 973.05 |
|  | Nonmixture cure model (log-normal) | theta | 0.0015 | NA | 710.80 | 722.13 | 0.0343 | NA | 960.52 | 971.90 |
|  |  | meanlog | 6.6960 | 4.9675 | 710.80 | 722.13 | 5.3261 | 2.7601 | 960.52 | 971.90 |
|  |  | sdlog | 2.1119 | 1.0114 | 710.80 | 722.13 | 2.0290 | 0.6820 | 960.52 | 971.90 |
|  | Nonmixture cure model (gamma) | theta | 0.5455 | NA | 710.68 | 722.01 | 0.4567 | NA | 961.43 | 972.81 |
|  |  | shape | 1.4772 | 0.3343 | 710.68 | 722.01 | 1.2702 | 0.2159 | 961.43 | 972.81 |
|  |  | rate | 0.0490 | 0.0481 | 710.68 | 722.01 | 0.0528 | 0.0382 | 961.43 | 972.81 |
|  | Nonmixture cure model (exp) | theta | 0.0000 | NA | 711.91 | 719.46 | 0.0969 | NA | 961.26 | 968.84 |
|  |  | rate | 0.0009 | 0.0022 | 711.91 | 719.46 | 0.0101 | 0.0176 | 961.26 | 968.84 |
|  | Nonmixture cure model (gengamma) | theta | 0.7456 | NA | 702.56 | 717.67 | 0.0237 | NA | 962.51 | 977.68 |
|  |  | mu | 3.0096 | 0.0105 | 702.56 | 717.67 | 5.5239 | 5.6059 | 962.51 | 977.68 |
|  |  | sigma | 0.0319 | 0.0481 | 702.56 | 717.67 | 2.1443 | 2.7760 | 962.51 | 977.68 |
|  |  | Q | 25.4523 | 38.1799 | 702.56 | 717.67 | -0.0575 | 1.2604 | 962.51 | 977.68 |
|  | Royston/Parmar spline model(0 kont) | gamma0 | -4.9999 | 0.4241 | 709.22 | 716.78 | -3.9851 | 0.2922 | 960.08 | 967.66 |
|  |  | gamma1 | 1.2659 | 0.1432 | 709.22 | 716.78 | 1.1245 | 0.1015 | 960.08 | 967.66 |
|  | Royston/Parmar spline model(1 kont) | gamma0 | -5.1719 | 0.6044 | 711.04 | 722.38 | -4.1875 | 0.3502 | 960.42 | 971.80 |
|  |  | gamma1 | 1.4156 | 0.3937 | 711.04 | 722.38 | 1.5667 | 0.3810 | 960.42 | 971.80 |
|  |  | gamma2 | 0.0375 | 0.0903 | 711.04 | 722.38 | 0.0430 | 0.0347 | 960.42 | 971.80 |
|  | Royston/Parmar spline model(2 kont) | gamma0 | -5.1471 | 0.6091 | 712.99 | 728.10 | -4.1659 | 0.3534 | 962.26 | 977.43 |
|  |  | gamma1 | 1.3656 | 0.4779 | 712.99 | 728.10 | 1.7224 | 0.5540 | 962.26 | 977.43 |
|  |  | gamma2 | -0.0790 | 0.4477 | 712.99 | 728.10 | 0.0841 | 0.1755 | 962.26 | 977.43 |
|  |  | gamma3 | 0.1750 | 0.7141 | 712.99 | 728.10 | -0.0660 | 0.2487 | 962.26 | 977.43 |
|  | Royston/Parmar spline model(3 kont) | gamma0 | -5.2573 | 0.6487 | 713.03 | 731.92 | -4.1589 | 0.3724 | 964.21 | 983.18 |
|  |  | gamma1 | 1.6440 | 0.5887 | 713.03 | 731.92 | 1.7590 | 0.6622 | 964.21 | 983.18 |
|  |  | gamma2 | 0.6410 | 0.5570 | 713.03 | 731.92 | 0.0610 | 0.3294 | 964.21 | 983.18 |
|  |  | gamma3 | -2.9374 | 2.1573 | 713.03 | 731.92 | 0.0244 | 0.7915 | 964.21 | 983.18 |
|  |  | gamma4 | 3.2026 | 2.3150 | 713.03 | 731.92 | -0.0819 | 0.7045 | 964.21 | 983.18 |
